# Unscripted Responsible Research and Innovation: Adaptive space creation by an emerging RRI practice concerning juvenile justice interventions

**DOI:** 10.1186/s40504-018-0066-1

**Published:** 2018-01-24

**Authors:** Irja Marije de Jong, Frank Kupper, Jacqueline Broerse

**Affiliations:** Athena Institute, VU University Amsterdam, De Boelelaan 1085, 1081 HV Amsterdam, the Netherlands

**Keywords:** Adaptive space, Responsiveness, Responsible research and innovation, Cognitive neuroscience, Juvenile justice institutions

## Abstract

Emerging RRI practices have goals with respect to learning, governance and achieving RRI outcomes (action). However, few practices actually achieve the action phase as actors lack room to manoeuvre, and lack guidance on how to move forward because of the inherent unscriptedness of the emerging RRI practice. In this explorative research an emerging RRI practice is studied to identify factors and barriers to the creation of adaptive space, in which actors can be responsive to the other and adapt, and a narrative can be created in the act of doing. This paper describes how formal and informal ways of organizing emerging RRI practices contribute to adaptive space, and how the metaphorical heuristic of improvisational theatre provides clear action principles to actors involved in emerging RRI practices in action. The RRI practice studied here lies in the domain of juvenile justice, where barriers that restrict room to manoeuvre are abundant. Five factors – ‘informality over formality’, ‘shared action space’, ‘be flexible’, ‘keep the action moving’ and ‘put the relationship central’ – were identified to facilitate reflexivity and adaptation in this space.

## Introduction

A key question to the governance of science and technologies in society is how to influence trajectories when negative impacts can be anticipated. Negative impacts can be technoscientific or macroeconomic in nature, but also ethical, social or cultural. As normative choices are being made throughout the entire research and technology development process and not only during the societal implementation phase, these aspects deserve consideration from the early stages onward. This is a key aspect in the emerging framework of Responsible Research and Innovation (RRI). RRI has been gaining traction from 2000 onwards in science policy in the EU and US as well as in the academic fields of policy and innovation studies (Owen et al. 2012, von Schomberg 2014). It stresses the importance of early inclusion of societal stakeholders, such as practitioners and social scientists, to enrich the research and innovation process from the outset (von Schomberg 2012).

Sites where this framework is being practiced have been emerging concomitantly, and can be placed into three categories according to an inventory by Kupper et al. (2015), each contributing to RRI in a different way: RRI practices for (1) learning, (2) governance and (3) action. Recognizing that irresponsibility in science and innovation is a derivative of the innovation ecosystem it originates from, RRI practices of the first category aim at opening up the science and innovation process to a wider range of voices to allow for collective learning among the different stakeholders. The intent of RRI practices for governance is for a new, more responsible direction to take hold, by ensuring that shared viewpoints and values emerging from such collective learning processes are reflected by the priorities set in the innovation ecosystem, and platforms are created to inform policy. Although the inventory by Kupper et al. 2015 identified plenty of emerging RRI practices of these two categories, examples of the RRI practices in action ‘doing’ responsible research and innovation to reach RRI outcomes, category three, are rare. For RRI products to arise, resources need to be spent on the actual solving of problems that have been inclusively defined, developing means to solve these problems by integrating different perspectives and knowledge traditions, and putting these means into practice in appropriate spaces while continuously reflecting on the process with a wider group of stakeholders. Emerging RRI practices for learning and governance seem to experience difficulties in transitioning to a phase of action. Our own activities aimed at creating and sustaining an engaged public (category one) produced potential solutions for policy problems, which were further explored via an agenda-setting process (category two), but then fizzled out (de Jong et al. 2016). Other multistakeholder experiences have also demonstrated that little action is found to follow from the inclusive deliberation processes typically found in RRI practices for learning and governance. In evaluation studies, this is often ascribed to incompatibilities with the incumbent regime (Arentshorst 2014, Hessels 2010, Kloet 2011, Roelofsen 2011). The new shared values and viewpoints are typically at odds with the dominant culture characterizing the innovation ecosystem. Action has been found to be constrained by formal and informal rules, regulations and procedures. Moreover, the dominant structure, culture and practice of the innovation ecosystem are resilient to change (Geels 2004, Roelofsen 2011) because of habits and routines, norms and expectations, or reward systems. In other words, the actors lack the means to be responsive to others and *adapt* their actions to acquired insights – resulting in changes in shapes or directions – as the incumbent structure leaves them no *space* to do so. In this paper we define this as the lack of ‘adaptive space’. If the actors were given the room to be truly responsive and adapt to the new insights obtained by the inclusive deliberation processes, an integrated scientific output could be achieved, which is a key element to RRI. Space can be social, material and geographical in nature and refers to an ability to manoeuvre; where plans can be adjusted following a progression of insight and where new members can enter at any given time (Rip and Joly 2012). In this space, people with different perspectives and backgrounds are present and have interactions with each other. At the same time, a space is delineated by its borders (that is: who is in and who is outside of the collaboration) and there are certain dynamics taking place within it which are internally arranged by the choice of structure or the rules and routines established (Rip and Joly 2012). These structures, rules and routines emerge from the interaction within the space and are in line with the shared values, viewpoints and ideas within the space, and are not merely a mashup of the structures, rules and routines characterizing the various epistemic cultures the participating stakeholders represent.

The goal of this paper is to explore factors relating to the creation of adaptive space to gain insights into how RRI practices in action can be created. The apparent importance of incumbent structures in inhibiting the emergence of RRI practices in action and their subsistence raises the question to what degree the creation and maintenance of adaptive space can benefit from formal and informal ways of organizing RRI practices in action. Moreover, we surmised that the inherent fluidity of emerging RRI practices heightens the challenge incumbent structures pose to emerging RRI practices in action. This fluidity concerns both the end goals of the RRI practice, as well as the collaboration within the practice. Mutual learning leads to new insights and possible changes in shape and trajectory, therefore concrete end goals are flexible. This mutual learning is taking place among collaborative partners who do not necessarily share a history of working together, then not only are the final goals open-ended, the collaboration should also be thought of as nascent and fluid. Moreover, changes in end goals can also require the initiation of collaboration with new stakeholder groups, or the leave-taking of others. Membership in the space is therefore also inherently in flux. One can easily imagine it is difficult to formally organize a priori for such ‘unscriptedness’. Rather, the end goals and the collaboration emerge from the act of doing in an iterative and adaptive manner. This paper therefore deals with two questions: (1) how to organize adaptive space with respect to the formulation of joint expectations, commitments for future action and coordinating executions of commitments in a formal and informal manner, and (2) how heterogeneous actors contend with the ‘unscriptedness’ of early collaborations. Below, we will elaborate on each of these points.

As a case study, we selected a collaboration between researchers and practitioners from juvenile justice institutions^1^ (JJIs) focusing on neurobiological measures for assessing the risk of recidivism and on implicit cognition in relation to substance abuse among detained juvenile offenders. This study is part of a wider research project on the responsible development and embedding of neuroimaging technologies,^2^ in which the first author is focusing on the domain of justice and security. The collaboration between researchers and practitioners from JJIs was selected as a case because adaptive space is particularly difficult to achieve in this type of collaboration, given the conflicting nature of the systems of science and of juvenile justice. Science aims at theoretical development and has its own set of rules and practices guarded by mechanisms such as peer review. The goals of the juvenile justice system, on the other hand, are more pragmatic and daily routines are heavily regulated and legally enshrined. To perform practice-oriented research in the heavily regulated and volatile environment of a juvenile justice system, adaptive space can be considered a requirement. The project will be described into more detail in the Case Description section.

### Organizing adaptive space

Since the 1980s, organizations have been increasingly forging relationships with other organizations, in alliances, partnerships or coalitions, to deal with or focus on new technological developments (Powell et al. 1996, Ring and Van de Ven 1994). Organization theories for studying these collaborations treat knowledge creation as inherently linked to concrete activities. These behaviourally based theories which assert the simultaneity of events and emphasise process (Cohen et al. 1972, Tsoukas and Chia 2002, Van de Ven 1986, 1993) are useful for collaborative practices within RRI. In the absence of well-defined goals, a well-defined technology and in a fluid collaborative setting, decision-making takes place under ambiguous circumstances. Interpretations of what participants are doing are then often derived from the experience of going through the process of doing (Cohen et al. 1972). Ring and Van de Ven (1994) have described the development process of such collaborative practices for cooperative inter-organizational relationships. This work indicates that when organizing for adaptive space, it is essential to realise that collective action is dynamic and goes through three temporal stages in a non-linear, iterative fashion: negotiations of joint expectations, commitments for future actions and executions of these commitments. Non-linearity means here that simultaneity of events is possible, in the sense that the *design* of an action does not necessarily *precede* the action, but can converge with it. This iterative approach can accommodate new insights - through mutual learning and collective action – for example by renegotiating commitments. Importantly, dynamic collective action is not only facilitated by formal approaches, but also informal approaches can be observed. Expectations can be negotiated through *formal* bargaining or *informal* sense making. Commitments for future action can be drawn up in *formal* legal contracts or commitments can be reached *informally* with a handshake (psychological contract). Executions of the commitments can be the result of *role* interactions or of *personal* interactions. By drawing on both formal ànd informal approaches, adaptive space can be observed to be created formally and informally. The former is more likely to provide stability to the adaptive space, the latter is more likely to contribute to its flexibility.

### Dealing with unscriptedness

So far, we have established that when concrete end goals are lacking, the how and why of the collaboration emerges from doing. The how and why of the collaboration can also be described as the ‘narrative’,^3^ which is being discovered while it is being played out. A concomitant feature of an emerging narrative, is emergent membership of the collaboration in the process of activity. In the process of doing, the collaboration learns about its own story but it at the same time discovers the players engaged in the story. This implies that the collaboration cannot be designed a priori following the end goals or the officially designated members. Rather, the emergent membership and the relations between these members take centre stage.

This unscriptedness is often overlooked in innovation literature. Recently, ‘intuitive’ decision-making is gaining increasing attention, especially with respect to this early phase characterized by high uncertainty (Dane and Pratt 2007, Eling et al. 2014, Sadler-Smith and Shefy 2004). In intuitive decision-making, hunches, gut feelings, subconscious and holistic associations are pivotal indicators of what decision to make, rather than a ‘good rationale’ (Dane and Pratt 2007, Eling et al. 2014). However, intuition as a concept does not yield a clear action perspective. For one, intuition is an individual process, and not a collective capacity (Crossan et al. 1999). Secondly, although intuition may guide individual action, it is difficult to share this intuition with others (Nonaka and Takeuchi 1995). This is due to the pre- or nonverbal nature of intuition (Cook and Yanow 1996). Bess and Dee (2008) have argued that these obstacles can be overcome by the use of imagery and metaphor. Therefore, we look here at improvisational theatre as a metaphorical heuristic. Improvisational theatre inherently carries within it the element of intuition, but has the advantage of developed *action principles*. By using it as a heuristic - rather than having the participants of the emerging practice engage in facilitated improvisational theatre exercises - it allows for studying ongoing conversations and actions in emerging RRI practices through the lens of improvisational theatre to make sense of facilitators and barriers in creating adaptive space. Such a heuristic also offers some accountability of the processes in which decisions have been made. Importantly, improvisational theatre emphasises collaboration in the absence of a pre-existing narrative. Rather, the narrative emerges through the act of collaboration.

Where traditional theatre has a script which dictates what the roles are, who plays what role, how the players interact, what objects are used, what the set looks like and where the story ends, improvisation is characterised by it being unscripted. Players are free to determine their own roles but also to change roles. The storyline emerges from the spontaneous interactions between the players as a collective activity. For their collaboration, actors are guided by some basic principles of theatrical improvisation that can be recognised in the work of Keith Johnstone (1979) and Viola Spolin (1999). These action-oriented principles can enable collaboration among members of a heterogeneous collaboration and allow for adaptation to each other and to changing circumstances. This potential for adaptation through improvisation is an important element of why improvisational capacity is being investigated in organization and marketing research, in the diverse applications of emergencies, (commercial or financial) markets and work organizations (Ciborra 1999). Because of their relation with adaptation, improvisation principles are thus interesting candidates for the creation of adaptive space in newly emerging collaborations. Examples of these action-oriented principles, to be found in the work of Johnstone, Spolin and others, are: to accept offers by saying ‘yes’ and build further upon what is offered to you; serving the good of the whole instead of the individual; being present in the moment, instead of being preoccupied with what happened in the past or trying to control the future; being open to change in your own ideas or in yourself in response to what is presented to you; looking for the personal connection, and taking risks (e.g. Johnstone 1979, Spolin 1999).

Importantly, improvisation can yield a narrative when all members of the team adhere to the mentioned basic principles. To do so, the players do not require prior knowledge of each other, nor do they need to be a homogenous group. In this way, it is an appropriate metaphor for the uncertain nature of early engagement with stakeholders without prior experience working with each other, and in the face of membership emerging from doing. Moreover, compared to concepts as ‘intuition’, it yields more guidance on how to act in this unscripted phase.

### This paper

While studying the collaboration, we paid attention to how the collaboration *organized* adaptive space − with respect to the formulation of joint expectations, commitments for future action and coordinating executions of commitments – and whether they used formal or informal strategies to achieve it. Secondly, we assessed whether attitudes or action-principles indicative of improvisation were present as a second way of potential adaptation to each other and to circumstances. From these observations, we distilled factors – facilitators and barriers – that influenced the creation and maintenance of adaptive space in this particular collaboration and may provide some guidance for other RRI projects in which adaptive space is desired to create responsiveness.

## Case description

The project in which we participated as an observer is part of an ‘Academic Collaborative Centre’ (ACC) in The Netherlands (see Table 1)(ZonMw 2015). These centres have been funded by The Netherlands Organization for Health Research and Development (ZonMw) since 2005. They are long-term partnerships between community health services, researchers and policymakers, to bring these practices closer together. The main purpose is to direct research activities towards grassroots level problems, and to implement the outcomes in practice. Within these ACCs (health care) issues are mainly addressed at the local level, instead of a national level. This means that problems felt by the practitioners are addressed and solved together with scientists and other professionals, and then implemented in practice. The experiential knowledge of the practitioners plays a fundamental role within these ACCs, and success of the ACC is not only measured in scientific terms, but in terms of the value of practical outcomes.

**Table 1:** Academic Collaborative Centres

These centres have been funded by The Netherlands Organisation for Health Research and Development (ZonMw) since 2005. They are long-term partnerships between community health services, researchers and policymakers, to bring these practices closer together. The main purpose is to direct research activities towards grassroots level problems, and to implement the outcomes in practice. Within these ACCs (health care) issues are mainly addressed at the local level, instead of a national level. This means that problems felt by the practitioners are addressed and solved together with scientists and other professionals, and then implemented in practice. The experiential knowledge of the practitioners plays a fundamental role within these ACCs, and success of the ACC is not only measured in scientific terms, but in tenns of the value of its practical outcomes.	

A particular collaboration in one of the ACCs focuses on the potential of applying neuroscientific knowledge and methods within JJIs. This site can be considered a promising RRI practice as it concerns an emerging collaboration between heterogeneous partners taking challenges at the grassroots as a point of departure. Although it is not an ideal RRI practice − for example, (former) juvenile delinquents should ideally have been included in constructing problem definitions, and there is insufficient attention to short-term feedback loops to allow deliberation on intermediary results in the on-going project − it does offer the opportunity to study a collaboration between scientists and societal stakeholders from the outset. This project is funded by the Dutch Ministry of Security and Justice (MoSJ) and combines two research projects: (1) testing the effectiveness of a computer training method for youth to cope with cannabis addiction by reducing implicit associations in the brain; and (2) investigating neurobiological predictors of juvenile recidivism (heart rate, measurement of hormones in saliva) to determine whether neurobiological measures can predict recidivism and whether these neurobiological predictors are of added value to the JJIs (see Table 2 for a description of the training programmes). The latter project has been assigned to PhD student A, the former to PhD student B. However, for the data collection for these two programmes to take place, the biggest challenge is the continuous intense logistical planning to get the adolescents to take part in the research. The researchers do not only have to contend with a number of safety regulations, but also with the highly regimented lives of the adolescents. Unlike adult detention, the youngsters do not remain in their cells for large parts of the day. Rather, their days are filled with strictly scheduled activities such as school, chores, physical activities, duties, disciplinary measures^4^ and privileges. This requires a lot of planning with a number of JJI professionals for each data collection event for each participating adolescent. Moreover, these negotiations took place in the process of getting acquainted with one another, which was another major goal of the researchers and the JJI professionals.

**Table 2:** Description of the training programmes

The training programme was originally developed to cope with alcohol addiction among adults in voluntary treatment, and was quite successful in that setting. It was because of a question by a JJI practitioner that this particular project was transformed for juveniles - and to include cannabis - and subsequently brought to that particular JJI. This practitioner felt that the low invasiveness of the training programme - short sessions behind a computer during which the participant pushes away and pulls certain images - would be suitable for the JJI context, as the adolescents are poorly motivated to confront their addiction.
A larger roll-out of this research happened after it was coupled to the second research component about neurobiological predictors of recidivism, by measuring heart rates and hormones in saliva. This research revolves more around a proof of principle and there is greater uncertainty on its benefit for the JJIs later on. It is noteworthy, however, that the research question emphasises the added value of neurobiological predictors compared with existing measures in the JJIs. If neurobiological measures can predict recidivism, the principle has been proven. They can still be deemed unsuccessful if they do not outperform existing measures in the JJI

The coupling of the training components described in Table 2 is efficient (they require the same basic information on the adolescents) and combines the attractiveness of a short-term gain (training programme) with the uncertainty of a long-term benefit (recidivism predictors). However, both deal with real-world challenges of practitioners within the JJI context. Substance abuse in justice institutions and decisions regarding release and penitentiary leave are highly politicised in the Netherlands.

The research takes place within the walls of the JJI, as the subjects are detained there. What is quite unique about this research is that it is truly taking place within each institution. To each JJI, at least two Master’s students are assigned, and stay there for the duration of about six months. PhD students A and B divided their time over the different involved JJIs (see Table 3 on the research team). It is not that these members of the research teams merely fly in and out to collect data; the JJI became the place of work.

**Table 3:** The research team

The research team consists of PhD students A and B, supervising researchers from their two respective universities and the Master’s students. PhD student A and some of the supervising researchers in the research team have experience with the psychological treatment of (troubled) children and adolescents. All have experience performing (neuro)psychological research. Practitioners of three JJIs were included in the early phase of the project, later the project was expanded to include more JJIs. Policy-makers within the MoSJ are also involved, albeit in an advisory capacity, and not on a daily basis. Focal point meetings take place at irregular intervals between members of the research team and the practitioners at the JJIs (varying between weekly and bimonthly depending on need). The researchers of the different universities meet each other amongst themselves on a monthly basis.	

## Methodology

### Data collection

Data was collected using multiple qualitative methods: observations of group meetings and site visits, informal conversations and semi-structured interviews (Bogdan and Taylor 1975, Hammersley and Atkinson 1983, Wolcott 1980). Furthermore, a log book was kept by the first author. Between October 2013 and January 2015, the sites of the researchers and five of the JJIs were visited (15 visits by the first author in total). The duration of visits varied between 1.5 h and 5 h. Furthermore, there was communication via e-mail and telephone with the various researchers. See Table 4 for restrictions during data collection.

**Table 4:** Data collection restrictions

The detained adolescents have a very strict day programme they need to adhere to. Each disruption of this routine creates extra logistics work for the personnel, as well as opportunities for other detained adolescents to seize to moment to create havoc. Because of the closed setting and safety precautions, including the privacy of the youth offenders, numerous logistical arrangements needed to be made. Furthermore, we were unable to interact with the juvenile delinquents themselves.	

#### Observations and informal conversations

The first author was allowed to join the PhD students on (certain) visits to the JJIs. During these on-site visits, the first author was present as a privileged observer (Wolcott 1980): a style of participant-observation where the researcher is familiar and allowed access to information while trying to be unobtrusive, with minimal interactions, observing the ‘business as usual’. Informal conversations with researchers, Master’s students and practitioners took place, with questions relating to observations and comments made by the researchers during the on-site visit at moments that did not disturb on-going interactions. This was important as the researchers of the ACC were quite protective of the limited time available to the practitioners at the JJIs. Observations of meetings and site-visits and informal conversations were included in a journal.

#### Timeline interviews

Timeline interviews were conducted with five participants of the ACCs research project: three researchers and two persons working at different JJIs. This provided a means for critical reflection and enhanced and deepened the perspective and meanings we had started to build during observations and informal conversations. In timeline interviews, interviewer and interviewee share the same large paper on which a timeline is drawn (Adriansen 2012). Key events for the collaboration are marked on the timeline. The interviewee can take ownership by drawing and writing, and influence the course of the interview. Nevertheless, the interviewer holds the final capacity to determine which issues are relevant or not. The method allows for different stories, different contexts, and different roles taken by the interviewee throughout the time period under discussion. The design of the timeline interview we used is presented in Table 5. The interviews were audiotaped after informed consent was obtained, and transcribed integrally.

**Table 5:** Timeline interview design

We asked after pivotal moments for the collaboration, good or bad, and let minor events emerge from elaboration on the major events. We were planning to be alert to elements of the process model by Ring & Van de Ven (1994) (negotiations of joint expectations through formal bargaining or informal sense making; commitments for future action through formal legal contracts or psychological contract; executions of commitments through role interactions or personal interactions) throughout the interview as they surfaced in the conversation. However, it turned out that asking after these elements (e.g. can you remember instances when expectations were made sense of?) was a good way for key moments to surface in the interview, as these elements were found to be particularly present in these key moments. After construction of the timeline, we reflected on the entire timeline by means of principles based on the metaphor of improvisational theatre. Different authors on improvisational theatre assert different ground principles. Based on the second author’s experience of being a performer of improvisation theatre himself and texts on improvisational theatre, we developed the set of principles shown in Table 6. Principles were written on post-its beforehand, and the interviewees were asked whether they felt that certain principles were applicable or not to certain events on the timeline. If so, they were invited to stick these principles next to the relevant occasion(s) and to explain why	

### Data analysis

The journal and the interview transcriptions were analysed using qualitative data analysis software (MAXQDA 11). The first author coded the journal and transcripts of the timeline interviews thematically. The codes represented principles of improvisation and the concepts of the model by Ring and Van de Ven (1994) (see Table 6). The coded segments were discussed by the first two authors and code categories were determined. Data analysis subsequently took place along identified themes. Results were discussed between the authors of this study as well as with members of the ACCs research project.

**Table 6:** Codes used during data analysis

Codes related to improvisation theatre		Codes related to the model of Ring & Van de Yen (1994)	
*(1) Yes, and ...* The golden rule of improvisation is to accept and build upon what is offered (Gesell, 1997).	*(4) Personal connections* Communication is not only a serious matter or useful and efficient to achieve targets. Communication can also be light and address the ordinary and aspects of everyday living.	*(A1) Informal sense making*	*(A2) Formal bargaining*
*(2) Make your fellow players look brilliant* The unified outcome is more than the sum of its part. This means being alert and willing to come to the other’s aid.	*(5) Be flexible* Willingness to change and/or let go of one’s agenda. Allow what is presented to you, to change you. Flexibility in trajectory and willingness to change roles for the good of the whole.	*(B1) Psychological contract*	*(B2) Formal legal contract*
*(3) Be present in the moment* Focus on and be receptive in the present moment, rather than being preoccupied by the past or the future.	*(6) Take risks* Keep the action moving, and step outside of your comfort zone by breaking routines. Dare to fail to do something new.	*(C1) Personal interactions*	*(C2) Role interactions*

## Results

Although the collaboration studied had not produced research outcomes by the time of observation, the collaboration was unanimously described as enjoyable and satisfactory. Despite the demanding conditions and the conflicting systems of science and juvenile justice, they were able to include a large number of subjects into the study, even though they faced the unforeseen closure of two of the five JJIs, with the accompanied drop in new placements of adolescents. Somehow, they were able to adapt to each other and to changing circumstances. Through the data-analysis we found that in this collaboration adaptive space was created by five main factors (see Table 7). Two related to *organizing* the creation of adaptive space: ‘informality over formality’ and a ‘shared action space’. Three factors were important for *maintaining* adaptive space: ‘flexibility’, ‘keep the action moving’ and ‘put the relationship central’. Each of these factors is described below.

**Table 7:** Identified themes in creating and maintaining adaptive space

Organizing adaptive space	Maintaining adaptive space
Informality over formality	Be flexible
- Preference for informal sense making, psychological contracts and personal interactions - Formality as life jackets	- Accept restrictions and be pragmatic - Stay in the present moment
	Keep the action moving!
	- Dare to be vulnerable - Be proactive
Shared action space	Put the relationship central
- Familiarity - Chance encounters and on-going activities	- Be empathic/generous - Sociability - Listening

### Organizing adaptive space creation

#### Informality over formality

Core aspects of collaboration development – negotiations of joint expectations, making and executions of commitments – started out somewhat formal, but quickly turned less formal. Initially, some formal bargaining took place between the researchers and policy-makers at the MoSJ, to acquire the earmarked grant. This was a formal contract, highly specific to the research that was going to take place and was accompanied with conditions, such as the frequency with which progress was discussed with officials of the MoSJ.

To recruit JJIs, supervising researchers plus one of the PhD students joined one of the regular meetings of the managing directors of all JJIs of the Netherlands. There, they explained their idea for the research and asked whether some of the institutions were interested in joining. The researchers did not have bargaining chips and they described this activity as ‘informing’ and ‘presenting’.

Three JJIs joined immediately, a few others lacked the capacity to join at that moment but joined later. Some formal contracts were signed at this point, such as confidentiality agreements and Certificates of Good Conduct (VOG, issued by the Dutch MoSJ declaring that the applicant did not commit any criminal offences relevant to the nature of the job). These are standard documents to sign when working, doing research or doing an internship at a JJI, and not specific to the nature of the collaboration.

Within the JJIs, negotiation of joint expectations initially took place along the hierarchy. At the start, the higher level of the JJI hierarchy and the supervising researchers were engaged in informal sense making of the opportunities of the research, the logistics and the required facilities during formal focal point meetings. Quickly, practitioners further down the hierarchy became more involved. As one researcher summarised it:

*We went down the organization layer by layer. So first the umbrella body, then the director and treatment provision chiefs of the individual JJIs, then chiefs of the groups, and then the group workers.*

Similarly, in later stages the supervising researchers were involved less, and the PhD and Master’s students played a bigger role. By the end of our involvement, most of the sense making activities took place at the lower level between the PhD and Master’s students, the group workers and some treatment providers during informal interactions. These chance encounters progressively became the site of most decision-making later on in the process.

The negotiation of joint expectations was simultaneous with the making of psychological contracts. Although some formal documents played a part in the beginning, most of the commitments concerned psychological contracts. These were principally verbal agreements. As one researcher put it:

*No contracts were drawn up, it was more in consultation with each other. Sure, we signed secrecy and VOG documents, but that’s standard paperwork. It wasn’t specific to this collaboration.*

By informal sense making of the opportunities of the research, the members of the collaboration started talking about how to facilitate the research, which led to making (verbal) agreements, which could lead to new questions about the nature of the research, for which further sense making was required. By talking about the logistics of the research – such as which room to test in and how to get the detained adolescents to the room, how to recruit and reward detained adolescents for cooperation, how to collaborate with group workers – they engaged in sense making and made the commitments at the same time.

Initially, the PhD students tried to formalise their interactions with the JJIs: they devised a plan who of the two was going to liaise with which JJI. This plan, however, was quickly abandoned in favour of letting this emerge from the process of doing. PhD student A came to liaise with all the JJIs. This was perceived to match the personal characteristics of PhD student A, a perception we shared. But circumstances also dictated the arrangement: it turned out that some of the research responsibilities required PhD student A to be at the institutions more often than PhD student B. PhD student B took on other responsibilities.

Gradually, conversations came to move fluidly between work and social talk. The researchers were quickly tapping into informal structures. For example, in one of the JJIs a formal contact person was appointed for their research (and ROM, see paragraph 4.2, put the relationship central). However, when this contact person fell away shortly after, the informal network was strong enough for this not to become a problem.

Contrarily, some activities were strategically *formalised*. In these instances, formality functioned as a ‘life jacket’ (Ring and Van de Ven 1994). Although the psychological contracts allow for high flexibility, at times some commitments were intentionally formalised by putting them in writing and for instance e-mailing it. This would allow these members of the collaboration to refer back to the written agreement should it be necessary in the future. Another illustration of the use of formality as a life jacket was when role interactions were strategically used. In case of imminent potential conflicts, higher placed officials in the JJIs were involved to correct the situation.

#### Shared action space

The locality of the research project was an important success factor of the collaboration. In collaborations between scientists and societal stakeholders, interactions are often located at the site of the *researchers*. This research, however, was taking place at the location of the *practitioners*. The practitioners favourably compared this research project with previous research projects where researchers were (far) less present at the JJI. The presence of the researchers meant that they were able to take up the logistical part of including the adolescents in the study, and that this burden was thus not resting solely on the shoulders of the practitioners. However, the locality was important for other reasons as well.

The PhD and Master’s students were physically present within the building of the JJIs. Besides the rooms for the experiments, they shared office space with JJI practitioners and were present in the group accommodations^5^ of the residing adolescents. Practitioners and members of the research team were involved in each other’s on-going activities. During the observations and the interviews, a frequent point was the necessity of the practitioners of the JJI and members of the research team being familiar with each other. In other settings familiarity may naturally and gradually arise as time passes. However, this is not the case for JJIs, as they are secured and closed settings. One practitioner said about this:

*Just over 200 people work here, in different groups and departments, and those are closed off from each other. So you can’t easily drop by another colleague like in a normal office. When you don’t need to be at a certain group, you really don’t know those people.*

Therefore, it takes a long time before employees of a certain JJI get to know each other. The same therefore goes for new researchers joining the JJI for a certain period of time unless the process is facilitated by the arrangements made for the collaboration.

In each JJI at least one of the two Master’s students are present at the institution. The PhD and Master’s students were often said to be the “face” of the research that other practitioners in the JJI needed to be familiar with, in order to start collaborating. If at any point in time someone within the JJI had a question about the research, someone would be available. Furthermore, PhD student A (and to a lesser extent PhD student B) travelled around all the involved JIIs and was therefore also part of the “face” of the research. This is quite different from regular collaborations, where researchers fly in and out to collect data, and can be reached via telephone or e-mail at their university. The members of the research team were experienced as easily accessible, which was deemed very important. On this aspect, this collaboration was also favourably compared to another previous research project by a practitioner.

The Master’s students were encouraged by both the practitioners of the JJI and by PhD students A and B to regularly visit the different accommodation groups and to spend quality time there, and to (re)introduce themselves regularly, especially because the practitioners work in shifts. Here, PhD student A lead by example by involving the Master’s students in PhD student A’s personal life as well. One researcher shared that the Master’s students:

*… cooked and had dinner with the group accommodation workers and detained adolescents or baked a cake for them to thank them for their cooperation.*

The fulltime presence of members of the research team allowed for flexibility in gathering the data, which is quite challenging considering the rigid yet often changing routine of the detained adolescents. But more importantly, the physical presence of the researchers at the JJI created opportunities for non-scheduled chance encounters and for interactions during on-going activities on a daily basis, to perform their respective tasks. Thus, besides the scheduled meetings that were prevalent in the early stages, it was *this* type of daily encounter that was of crucial importance for the perceived success of the collaboration.

### Maintaining the adaptive space

In organizing adaptive space creation, a shared action space thus seems important, as well as a preference for informal collaboration structures with the strategic use of formality as ‘life jackets’. In this section we explain which actions aid in *maintaining* the adaptive space. These can be interpreted as behavioural guidelines that can be followed during daily activities, but they may also be understood as personal attitudes.

#### Be flexible

Although there are strict routines for adolescents within the JJIs, their availability can also be unpredictable, for example when an adolescent has received a disciplinary measure or an incident has happened at the group. Both aspects make it difficult to plan research activities. As one practitioner put it:

*When you set foot in the door, you never know what your day is going to look like.*

It is therefore imperative to be flexible, which is seen by the interviewed members of the collaboration as a core attitude. One researcher said:

*If you’re not flexible in [practice-oriented] research, you just shouldn’t consider it.*

This indicates that flexibility is not merely an action, but can also be understood as an attitude. The basic attitude of the researchers was to be flexible *themselves*. One researcher put it:

*We are not merely expecting them to make room for us.*

They accepted restrictions in the JJI, and chose to adapt to it in a way that is still acceptable for the rigour of the research, but less intrusive to the practices in the JJI. As one practitioner put it:

*I notice that [the researcher] is trying to think of new ways and shifting things around, but at certain points [the researcher] needs to stand firm, which I understand.*

For example, to cope with the restriction in available time with the detained adolescents, the researchers replaced the long IQ questionnaire with a short working memory test as a derivative. As each JJI had their own restrictions, the research routines evolved differently as well, but without endangering the integrity of the overarching research goals. For example, time slots in which the adolescents could be tested were adjusted to the situation of the particular JJI. Similarly, compensation for participation by the adolescents in the research project varied because of tailoring to the JJI rules and culture, as well as to the specific population in the JJI.

The researchers thus seemed to anticipate and even *accept* inflexibility on the part of the practitioners, considering the heavily regulated environment of the JJI. But also the JJI practitioners showed flexibility, for example by changing parts of the daily schedule to accommodate the work of the researchers. The researchers seemed to appreciate flexibility on the part of the practitioners as a gift: they did not expect nor command it in trade for their own flexibility.

Flexibility was also achieved through interactions between a researcher and JJI practitioner to adjust the plan. As one JJI practitioner put it:

*But at this point we discovered, it is not working. We are getting stuck. So we had to be flexible and [together] come up with a new way.*

An important part of being flexible was to stay in the present. Certainly, sense making took place on the goals of the research and on values underlying the research. For example, concerns about reductionism when using neurobiological measures were discussed in one of the first interactions between researchers and JJI practitioners. Nevertheless, most of the communication in the interactions concerned the daily state of affairs. It was not that medium-term plans and considerations were absent, but they did not surface in the interactions between the heterogeneous partners. For example, at one stage the researchers were thinking of adding a new component to the research. Although this was discussed by the researchers amongst themselves, it did not surface in the conversations with the JJI practitioners. In the end, the addition of the new component was not in fact proposed, as it was difficult to realise and lacking in added value considering the cost. One researcher explained this in the following way:

*Certain things are for a later stage [to be discussed]. I often notice that when things have been talked about in detail before, once it becomes relevant, circumstances can have completely changed. And then nobody remembers anymore what was discussed before. So it is better to [stick to the moment] and to assess if things are still going well.*

Another telling example was when shortly after deciding to join the research, one of the JJIs heard that it was scheduled to close, although it was uncertain when exactly. Instead of opting out, as the research was less likely to yield benefits for this JJI specifically, they decided to continue. Practitioners from this JJI said about this:

*As long as we’re not closed yet, we will just get to work [with this research].*

However, institutional barriers considerably constrained flexibility. Regulations within the JJIs were recurrently at odds with scientific practice, one example was that of time needed to test the subjects to academic standards versus the strict schedules of detained juvenile offenders. We observed that the earmarked funding prevented new research questions from being taken up in the project. Although the researchers coped with this by keeping a log of questions to consider as new projects within the wider ACC, having an earmarked fund did limit possibilities for emergent research design. The same was true for institutionalised academic and clinical ethics. The juvenile delinquents themselves were little involved. For example, they were able to influence the timing of their participation, but they were not involved in sense-making on the purposes of the research. At the beginning of the first author’s involvement in the project, her inclusion sparked the discussion on whether it would be possible to include juvenile delinquents in group discussions on research purposes and choices. Despite favourable attitudes, this was considered too complicated, because it would require a resubmission of the research to the medical ethical committee, which would mean a long delay.

#### Keep the action moving!

Engaging in practice-oriented research is not without risks for the parties involved. For the JJIs, being involved in research to decrease substance abuse can draw attention to problematic drug use among juvenile delinquents in general, but also to the possibility that problematic drug use continues within the walls of the JJIs. The researchers trying to find out whether neurobiological measures have an added value to existing methods of assessing recidivism, can find out that their scientific method may be sound but yet not of added value. Researchers thus make themselves more vulnerable to outcomes which are not in line with their scientific discipline (or career path). One researcher said about this:

*It is possible that the outcome of this research is that neurobiological measures are not predictive enough. And then you [have to] say: Let’s stop this [line of research] and focus on other things.*

These risks need to be accepted to keep going anyway. The researchers themselves expressed that they needed to be proactive, but this was also expected of them by the JJIs. One practitioner said:

*[The researcher] took matters into her own hands, which is really necessary within the institution (…) At the start of the research I thought to myself, let [the researcher] figure it out for a while, and see how far [the researcher] gets (…) and that went really well.*

But also the culture of JJIs was being described as proactive. One researcher put it:

*Well, that’s their mentality, going forward against all currents. I think you really need that mentality if you’re working with delinquents.*

#### Put the relationship central

As an observer, it was fascinating to see how much energy was put into building the relationship between researchers and JJI practitioners. The main characteristics of this process were empathising, socialising and listening. During the first meeting at one of the JJIs, both JJI practitioners and researchers empathised clearly with the other. Each party continually raised points they thought the other would care about and suggested solutions for those particular problems. During conversations between the first author and the researchers, the researchers often emphasised the need to make things easy for the JJI and to avoid getting in their way as much as they possibly can. One researcher put it like this:

*From the start, our approach has been to make it as easy as possible for them for letting us do the projects there. One of the key points has always been to burden the institution as little as possible.*

A crucial moment was the renegotiation of the involvement of the Master’s students in the Routine Outcome Monitoring^6^ (ROM). ROM was about to be implemented in all JJIs by order of the MoSJ simultaneously with the start of the ACC project described in this paper. To make it easier for the JJIs, the researchers offered that Master’s students would help in collecting data for ROM. However, when it turned out that the implementation was going to be delayed and that detained adolescents were reluctant to cooperate with the research team when it concerned data that was going to be shared with the JJI, renegotiations took place. At that time, the relationship was strong enough to survive this.

The JJIs were quite generous in arranging facilities for the researchers and the freedom that was allotted to them. Some practitioners mentioned that this was more the case now than in certain previous research projects with other researchers. Furthermore, researchers showed their involvement in the sense that they cared about the group of juvenile delinquents and that they understood what it means to work with them. This made working together easier. As one JJI practitioner put it:

*[The researchers] care about the topic, the target group and the JJIs. And therefore there is a lot more intrinsic motivation to shape this research together.*

In this part of the conversation, this practitioner favourably compared this research project to another research project where the commitment of the researchers was far less present.

Also during training sessions, Master’s students were encouraged by the PhD students to show their interest in the JJIs, for example during the first visit of the JJI that was going to become their place of work for the coming period of time. One PhD student also expressed the importance of forming a personal connection with the Master’s students ànd the JJI practitioners and said:

*I notice that it motivates the Master’s students to [form personal connections] in their institution. They stop by for a cosy chat with group workers and the adolescents, and I encourage them to do so. It makes doing the research so much easier (…) Now [that I’m dividing my time over more JJIs] I notice that when I’m there, the connection is good (…) but I don’t think we should add more JJIs. Then it won’t work anymore.*

The other interviewed researchers and JJI practitioners also acknowledged the importance of social interactions for smooth and enjoyable collaboration: making jokes was one of the examples mentioned. Note that expansion of the research to other JJIs is deemed unfavourable to the collaboration, even though expansion would make it easier for this particular researcher to achieve the number of included adolescents needed for statistical calculations.

Practitioners also emphasised the importance of the researchers being prepared to listen, rather than to preach. Previously, there had been experience of students immediately talking about what should be changed, based on what they had learned in the school benches, without really asking why things were as they were. They expected that the research team should look around, observe, get surprised, wonder, and most importantly, ask questions. In postponing judgement, they might learn that things may be different than they appear. Researchers were also observed by the first author to listen during the collaboration. For example, when practitioners raised alternative research topics that would be of use to them. They kept a list for future reference although, as the funding was earmarked, they were not able to incorporate it in their research directly.

## Discussion

### Delineating the adaptive space

The members of the collaboration were able to adapt in several ways: they adapted to changing external circumstances (e.g. the announced closure of two of the participating JJIs and the concomitant drop in placements of juveniles), because of progressive insight (the difficulties with respect to the ROM), and to accommodate diversity between the different participating JJIs (in each JJI its own routine emerged, without endangering the integrity of the overarching research goals). However, the room to manoeuvre was restricted and the border of the adaptive space was rather rigid. The border was mainly constituted of institutional barriers, the most important being the legal and regulatory restrictions within JJIs, the institute of science, the nature of earmarked funding and institutionalisation of academic and clinical ethics. Within these borders, they were able to maximise room to manoeuvre and create new joint routines.

We observed several facilitators and barriers of adaptive space creation and maintenance, which will be discussed below. The identified non-institutional barriers largely relate to anecdotal evidence where the collaboration studied here was favourably compared to other previous research projects in collaboration with JJIs.

### Facilitators and barriers for heterogeneous collaboration

Emerging heterogeneous collaborations are generally not smooth or always enjoyable. In wider literature, facilitators and barriers for heterogeneous collaborations such as those taking place in emerging RRI practices can be found in literature on transdisciplinarity (Thompson Klein et al. 2001). Core characteristics of RRI strongly resonate with those of transdisciplinary research (Wickson and Carew 2014). They share a focus on multidimensional real-world problems, collaboration and mutual learning between heterogeneous researchers and societal stakeholders and iterative processes (Wickson and Carew 2014). Facilitators and barriers for these (transdisciplinary) collaborations are often discussed on the levels of the intrapersonal, interpersonal, institutional and physical.^7^ For clarity, we have put theresearch themes (as displayed in Table 7) in italics as they come up in the discussion. An overview of the identified facilitators and barriers for each level is displayed in Table 8.

**Table 8:** Facilitators and barriers of adaptive space creation and maintenance

	Facilitators	Barriers
Intrapersonal	- Collaborative attitude. - Commitment. - Be flexible, in the sense of accepting restrictions and be pragmatic in the face of them. Stay in the present moment. -Keep the action moving! Dare to be vulnerable and be proactive.	- No shared commitment. - No willingness to be flexible. - Living in the past/future. - Waiting for the other to take initiative. - Lack of willingness to accept risk.
Interpersonal	- Put the relationship central, by being empathetic (listening to the other and anticipate the other’s need), being generous (make things easy for the other by anticipating the other’s needs and by being easy to reach) and investing time in forging personal relationships (small talk, humour). - Flexibility with respect to methodologies and changing circumstances. - Informal negotiations of joint expectations.	- Researchers are difficult to get hold of. - Lack of time invested in personal relationships. - Feelings of entitlement, rather than generosity, e.g. expectation that the other will accommodate and anticipate your needs. - Focus on educating the other, rather than listening to the other. - Sticking to one’s own methodologies. - Failure to let new routines emerge.
Institutional	- Informality over formality: preference for informal sense making, psychological contracts and personal interactions. - The use of formality as life jackets.	- Focus on formal contracts to formally arrange adaptive space. - Institutionalized academic/clinical ethics. - Earmarked funding of research.
Physical	- Shared action space: to create familiarity and facilitate chance encounters in which on-going activities can be discussed.	- Heterogeneous partners that do not share a space (part-time or full-time). - Interactions take place during formal focal points only. - Lack of familiarity.

#### Intrapersonal

Two important facilitators of the collaboration studied was the willingness of the members to be *flexible* and by *keeping the action moving*. The members were *flexible*, without compromising their own integrity or that of their respective institutions (i.e. justice and science). Importantly, the researchers accepted the restrictions within the JJI and were flexible without expecting the practitioners to be flexible as well. Another source of flexibility was the focus of the collaboration on the present moment in their communications and a permissive attitude towards ambiguity on activities considered for the medium term. Circumstances can change in the meantime, and there is the possibility of members of the collaboration growing towards each other, without forcing the other to take a position on something that might not even become relevant in the future. This type of flexibility resembles the concept of ‘collaborative readiness’ (Hall et al. 2008, Stokols et al. 2008), which conveys the team members’ preparedness for the uncertainties and complexities inherent in transdisciplinary teamwork.

The notion of *keeping the action moving* corresponds with one described characteristic of ‘great groups’ (how Bennis (1997) calls it), being a sense of urgency and a corresponding willingness to risk failure. Furthermore, it is striking to see how much the researchers were committed to perform practice-oriented research and to the challenges of the JJIs. Anecdotal evidence from the JJI practitioners suggest that the lack of this can create a significant barrier. Previously, Roelofsen (2011) found that commitment is crucial for intended activities formulated through multi-stakeholder session to consolidate into real action. In a previous heterogeneous collaboration project our research group was involved in, 18 partnerships between arthritis researchers and societal partners (patients) were monitored, of just three survived at the two-year mark (Elberse 2012). There were some notable differences between these two projects, for example, collaboration with professionals versus patients and the presence or absence of a structured environment in which collaboration can take place. For the arthritis project, it was the intention that a new structure, practice and culture was to be established at the site of the *researchers* during the collaboration, whereas in this study the collaboration took place in the non-academic setting of the *practitioners*. Interestingly, in the successful partnerships of the arthritis project researchers recruited societal partners themselves (instead of waiting for the project coordinator to assign them). This move towards the ‘other’ therefore seems important in the arthritis study as well as the one described in this paper. Furthermore, in successful collaborations, more proactivity was observed and more face-to-face meetings were arranged. This points towards the necessity of a certain collaborative attitude.

Our study results remain ambiguous with respect to the nature of the identified factors. Are the factors of ‘flexibility’ and ‘keeping the action moving’ action principles or attributes? We saw that members acted according to these principles, but that they sometimes differed in personal abilities and attitude. For example, one of the members of the research team expressed more instrumentalist reasons to act in a certain way, while for others the action seemed to emanate from an embedded attitude. More research is needed to uncover the nature of these factors: will the factors continue to be linked to successful collaboration if they were to be part of a formal protocol? Or do you need persons who have certain attributes?

#### Interpersonal

Much time was invested in the *informal* negotiation of joint expectations with practitioners throughout the entire institution. This resonates with the wider held belief that the process of making expectations explicit and development of shared visions or goals are crucial success factors for transdisciplinarity (Cooperrider and Srivastva 1987, Kayes et al. 2005). The above described facilitator of collaborative readiness (Stokols et al. 2008) also includes openness to other disciplinary perspectives and world views, as a willingness to invest substantial amounts of time and efforts to the building of relationships. *Putting the relationship central* was an important facilitator of the adaptive space studied here. Both researchers and practitioners tried to make it easy for the other. Importantly, the researchers did not expect to be accommodated in such a way. Rather, they were generous with their time with respect to being easily accessible and investing in the formation of interpersonal relationships. The latter is also a recognised element in enhancing the success of transdisciplinarity (Creamer 2004). The fact that researchers listened to what practitioners had to say instead of trying to educate the practitioners how to do their job was one of the facilitators. By contrast, during a heterogeneous encounter between neuroscientists and educational professionals, a connection between the stakeholders failed to be made in large part because scientists focused more on educating the professionals on ‘good education’ than on listening to them (Edelenbosch 2014).

In the collaboration described in this paper, team roles emerged organically, showing a preference for *informality over formality*. Given that the early phases of heterogeneous collaborations are characterised by high uncertainty, and possibly ignorance, collaborations should be approached as processes that build long-term relationships. Furthermore, members of the collaboration should be able to adapt *flexibly* to the changing circumstances and with respect to methodologies (Israel et al. 1998, Stokols 2006, Stokols et al. 2008) and to let new routines emerge.

#### Institutional

For the creation of adaptive space, *informal* Strategies were preferred over formal strategies in this collaboration. Although they fulfilled existing *formal* obligations (signing standard forms for collaboration) and organized formal meetings, the structure had no formal hierarchy. This allowed for *flexibility*, so that the members of the collaboration could focus on the relevant problems at hand. Furthermore, it allowed for varying levels of membership - between peripheral and full participation - as the specific problems required at that certain moment in time. Occasionally, they used formalisation strategies as life jackets.

Possibly, the preference for *informal* strategies is prompted by the high degree of barriers this space is inherently facing. Besides legal and regulatory restrictions within the JJIs, science has a high degree of institutionalisation and the practice of science is regularly at odds with the goals of the JJIs. Furthermore, the nature of the earmarked funding and the institutionalisation of academic and clinical ethics made it difficult for the members of the collaboration to shape the research in an emergent fashion. RRI practice is thought to require emergent design (Wickson and Carew 2014), which is not easily compatible with how academic and medical ethics committees work.^8^ Nevertheless, new routines for the execution of the research have been established within the JJIs, indicating that some institutionalisation is taking place. So far, they have not moved towards high standardisation and institutionalisation. Institutionalisation offers the benefit of stability (Rip and Joly 2012). With respect to the outside world, the wider ACC structure funded by ZonMw lends some legitimacy to the existence of the heterogeneous collaboration studied here, thereby also stabilising the space. It is questionable whether deeper institutionalisation would be attainable or even desirable for this particular space. Considering the high degree of institutionalisation in both justice and science, it could be that emerging spaces at their cross-sections can only survive without formal commitments. Kessel and Rosenfield (2008) have, for example, argued for non-hierarchical arrangements for transdisciplinarity, because of the constraints associated with rigid hierarchic structure. Similarly, Stokols et al. (2008) contend that non-hierarchical organizational forms of transdisciplinary collaboration support inclusiveness and maximises collaboration. Moreover, informal approaches have recently also been found in similar collaborations between scientists and companies in the highly commercialized domain of global pharma (Morrison 2017), indicating that informality is not restricted to collaborations characterized by a low degree of market pressure, such as the collaboration described in this paper. Although two of the five JJI’s involved in the collaboration described here were privately owned – the three others are under government control – JJI’s mainly cater to specific regionally designated areas, meaning that competition is relatively low compared to most commercial organisations. Morrison also noted that a certain degree of *familiarity* was a prerequisite for informal approaches, which is in line with our findings (see below).

More research would need to take place, for example to see whether this adaptive space will remain informal or whether deeper institutionalisation will take place in later phases. In particular, the emergence of research outcomes would be an interesting phase to study, as this could give another dimension to the ‘success’ of this collaboration in relation to the adaptive space that has been created. So far, we have been assessing the success of the collaboration in terms of the inclusion of subjects into the research and the experience of the individual members of the collaboration.

#### Physical

An important organizational prerequisite for the creation of adaptive space was the development of *familiarity through a shared action space*, where chance encounters could take place and on-going activities could be adjusted. The relevance of face-to-face contact has also been put forward in studies on transdisciplinary collaborations of spatially dispersed teams (Lipnack and Stamps 1997, Olson and Olson 2000). Stokols (2006) argues that team members’ spatial proximity is a key facilitator of transdisciplinary collaboration as it encourages *informal* contact and communication. A shared action space as described in this paper provides the opportunity for regular and unconstrained interpersonal and project-related communication, which is a precondition for the establishment of trust and clarity concerning joint expectations and roles (Stokols et al. 2008). In the project with the arthritis researchers described above, the lack of a shared space was experienced as a barrier. Most research decisions were made ad hoc via *chance encounters*, for example at the coffee machine. As the societal partners had to be specifically invited, they could not take part in these day-to-day processes.

### Methodological discussion

The identified factors shaping adaptive space emerged in setting characterised by a high level of regulations and legal restrictions and a highly politicised subject matter. However, the factors seem universal enough to be relevant for collaborations in other settings as well. It could be that the complex circumstances allowed these factors to come into sharp focus. More research will be needed to investigate the generalizability of these factors. We have not been able to observe the collaboration in all its phases, which could also yield different insights into the factors relevant to the creation of adaptive space. Furthermore, we were unable to talk to the detained adolescents, or to observe their interactions with the researchers. This was not possible due to the protection of their privacy and due to time limitations, however, their input would have been valuable. The authors are nevertheless grateful for each opportunity given to join the ACC’s research project and visit the JJIs to observe. Formal interactions with JJI personnel were sparse. The primary task of the personnel is already a strenuous activity. The execution of scientific research in such facilities is considered to be a demanding secondary task. Let alone the presence of another scholar, the first author, doing meta-research in a project that is a lot less likely to deliver tangible outcomes for the facility itself.

### Responsible research and innovation

The collaboration described in this paper was achieve a sustainable practice of action. At the moment of writing they have been active for over 3 years. The theory on communities of practice provides a relevant perspective on how they achieved sustainability (Wenger 2000). We will discuss the indications that a community of practice was formed around the research experiments of the ACC through the employment of the factors facilitating the creation and maintenance of adaptive space. In the JJIs, mutual engagement is taking place between members of the research team and practitioners of the JJI in the absence of a formal structure. Their interactions revolve around the research experiments, which are being shaped by collective negotiation of meaning by the members of the collaboration. This has so far resulted in the development of a shared repertoire: the development of routines and a shared language. Examples of the latter are descriptions of ‘making things easy’ for the other, as well as the formulated need of the research to have a ‘face’. Through their interactions, the members of the heterogeneous collaboration learn how to shape such research experiments within the walls of the JJI and they shape the process of the research together. In that sense, the research process is a shared process that allows the members to coordinate their actions across the boundaries of science and JJI. As such, the research process can be considered a boundary object (Star and Griesemer 1989) within an emerging community of practice. Importantly, it is within such communities of practice that people acquire knowledge and give meaning. As the production process of the emerging routine was highly contextualised – it took place in a shared action space – the routine as an outcome of this process can be considered as ‘socially robust knowledge’ (Nowotny 1999). Fitzgerald et al. (2014) have recently also explored the neuroscientific experiment as a mode of knowledge production. The knowledge base resulting from the shared shaping of the research experiment, can be used to further develop and refine the current research experiment, but also future experiments. Hence, the knowledge generated in this heterogeneous collaboration does not only concern the content of the current collaboration, but can also include knowledge that contributes to furthering methods of practice-oriented research more generally.

The results of this paper therefore show that the emergence of a community of practice creates opportunities to translate imaginaries and plans into ‘doing’. RRI practices in action require a high degree of on-going interaction and close proximity. Continuity seems attainable by gradually constructing a shared action space, where informal, personal and day-to-day interactions can take place. The adaptive space can be maintained by adhering to action principles. This can gradually lead to the shaping of a community of practice. Although communities of practice are evolving, in the sense that membership and routines can change over time for example, they are essentially sustainable. Therefore, concerns of continuity are less for RRI practices in action which have been successful in creating a community of practice. However, as we have also observed, RRI communities of practice are likely to run into institutional and wider systemic barriers. Therefore, the development of the community of practice may benefit from ‘reflexive monitoring in action’ by a monitor who assesses progress and initiates reflexive deliberation on these barriers and strategies to overcome them (Van Mierlo et al. 2010).

The metaphor of improvisation also provides a new utensil for the toolkit for RRI. Results of previous evaluations on organizational improvisation suggest that improvisation is linked to emergent learning (Mintzberg 1996) that can be strategically employed as a substitute for planning (Weick 1987). In that sense, improvisation is an appropriate metaphor for the RRI characteristic of ‘responsiveness and adaptive change’ (Klaassen et al. 2014), which is further supported by the outcomes of this study. Furthermore, compared with the notion of ‘intuition’ with its indicators ‘gut feeling’ and ‘hunches’ located at the subconscious level, the action principles within improvisational theatre have a great advantage both for researching the early collaboration phase, as well as for shaping adaptive space. Principles such as ‘flexibility’, ‘keep the action moving’ and ‘put the relationship central’, are easier to observe as a social scientist than processes at the subconscious level of the participating members. As a metaphorical heuristic it thus offers benefits. Moreover, the action principles also offer benefits to the members (or managers) of such sites. Within the ACC project, we observed for example how a certain behavioural rule – making things easy for the other (action principle ‘put the relationship central’) – emerged, became part of the shared language and was embedded in the routine. Transmission and adoption of concrete action principles is probably more straightforward than empowering members to act on hunches at the subconscious level.

## Conclusion

Shepherding the evolution of heterogeneous collaborations in emerging RRI practices, by nurturing the relationships between the members and investing in long-term bonds, appears to be possible in the absence of a structure consolidated by formal commitments. Collaboration seems to get better as: researchers spend more time at the location of the practitioners; proximity between science and practice increases (for example when researchers show involvement and are attentive to knowledge questions emerging from the practice); researchers are more proactive and easy to reach. Spaces will emerge from transactions between heterogeneous stakeholders (Rip and Joly 2012). The challenge is to configure the space in such a way that reflexivity and adaptation is possible within the constraints the space necessarily faces. The five factors we described here can help in creating and maintaining adaptive space.

## Abbreviations

ACC: Academic collaborative centre
JJI: Juvenile justice institution
MoSJ: Ministery of security and justice
NWO: Netherlands organisation for scientific research
ROM: Routine outcome monitoring
RRI: Responsible research and innovation
VOG: Certificate of good conduct
ZonMw: The Netherlands organisation for health research and development

## Footnotes

1 Juvenile justice institutions are facilities where juvenile offenders are placed in legal custody, where they may be treated for behavioural problems and attend schools that are attached to these institutions (mostly vocational training).

2 Neurosciences in Dialogue aims to involve researchers and societal stakeholders in an interactive learning process to stimulate developments of neuroimaging technologies towards shared desirable applications with few, or at least manageable, negative impacts in society. It focuses on three application domains in particular, being medicine, education and justice & security. The first author of this paper is concerned with the latter application field.

3 A narrative refers to the way events are ordered in a meaningful whole. More specifically, in an iterative process characterizing RRI, these events and meaning are not known a priori, but jointly discovered while or after the event has taken place. This narrative subsequently informs future actions and interpretations.

4 When having broken a rule, the adolescent or the entire group the adolescent is part of, can receive a disciplinary measure, such as taking away a privilege or confinement in their bedroom.

5 The group accommodations usually house about four to ten adolescents, depending on the availability of placements and the (psychiatric) issues the adolescents are dealing with. The group accommodations are furnished like a home, and the adolescents all have a room of their own within this group accommodation.

6 Routine Outcome Monitoring concerns the repeated measurement of characteristics of the juvenile offenders, with the aim to evaluate changes in the course of time, allowing for estimations of benefit from treatment in the institutions.

7 The technological and socio-political level are also recognized, for example by Stokols et al. (2008). We will not discuss these here, as they were not as readily identified as the other levels.

8 This is a wider concern. See the Sussex Conference ‘Have We Become Too Ethical? Managing vulnerability in human subject research’ which took take place on November 9, 2015. www.centreforbionetworking.org
